# Is prevention better than cure? A systematic review of the effectiveness of well-being interventions for military personnel adjusting to civilian life

**DOI:** 10.1371/journal.pone.0190144

**Published:** 2018-05-02

**Authors:** Andreas Bauer, Dorothy Newbury-Birch, Shannon Robalino, Jennifer Ferguson, Sarah Wigham

**Affiliations:** 1 Institute of Neuroscience, Newcastle University, Newcastle, United Kingdom; 2 School of Health and Social Care, Teesside University, Teesside, United Kingdom; 3 Institute of Health and Society, Newcastle University, Newcastle, United Kingdom; Uniformed Services University of the Health Sciences, UNITED STATES

## Abstract

Exposure to stressful and potentially traumatic experiences is a risk for military personnel and for some this may increase susceptibility to reduced well-being. The aim of this systematic review was to examine the effectiveness of interventions to promote the well-being of military personnel adjusting to civilian life. Electronic databases were searched including MEDLINE, Embase, HMIC, PsycINFO, Pilots and CINAHL. Twelve articles, all conducted in the USA, were included in the review. Articles were synthesised narratively and assessed for bias against established criteria. The studies evaluated the effectiveness of interventions for current and former military personnel. The interventions included expressive writing, anger management, cognitive training, psycho-education, and techniques to promote relaxation, connection in relationships and resilience. Interventions had some significant positive effects mostly for veterans adjusting to civilian life and other family members. There was much heterogeneity in the design and the outcome measures used in the studies reviewed. The review highlights the need for future robust trials examining the effectiveness of well-being interventions in military groups with diverse characteristics; in addition qualitative research to explore a conceptualisation of well-being for this group and the acceptability of interventions which may be perceived as treatment. The results of the review will be of interest to a number of stakeholders in military, public health and mental health settings.

**PROSPERO Registration number:** CRD42015026341

## Introduction

The stressors experienced by military personnel during deployment are different and potentially more traumatic than encountered by many people, and for some these high risk experiences may have an impact on well-being [1–3]. In addition, research around transition and adjustment to civilian life indicates that leaving the service may also be a challenge to well-being as military personnel and their families can be required to manage a number of simultaneous life changes around occupation, finances, identity, social support networks and relationships [4,5]. Similar adjustments may be required post-deployment, and may occur over a period of time after leaving service, and could be ongoing for Reservists. The cumulative effects of these changes and required adjustments have the potential to increase stress and for some may be a challenge to well-being [6–8].

The concept of well-being is broad and multi-dimensional and has been defined as comprising subjective and objective elements of mood, emotion, life-satisfaction, social and psychological functioning [9–11]. For example, balancing a sense of purpose and meaning in life, with autonomy, healthy relationships, and good social support are suggested to be important for protecting against psychological distress and maintaining mental health and well-being [9–11]. The World Health Organisation (WHO) equate mental health with well-being whereby people are able to cope with daily stressors and use their abilities to work and contribute to society [12]. Well-being can be conceptualised as a continuum rather than a dichotomy in the manner of clinical diagnoses and may be compromised by stress or distress that does not meet diagnostic thresholds or come to the attention of clinical services, so there may be reluctance or no requirement to seek professional help [13–17].

Ways of coping with stress or distress however may compromise well-being by causing sub-clinical difficulties; for example, susceptibility to substance misuse or risk-taking has been found after deployment [18–22]. This may cause further negative life events for example by adversely affecting relationships or compromising the ability to function optimally, and so reducing quality of life [20]. Therefore, the well-being of military personnel and their families may be impacted even though clinical thresholds are not met and specialist clinical care is not required or sought [18–22].

Preventative early interventions to protect well-being may facilitate the development of self-help ways of coping with stress or distress, and may be implemented upstream of clinical services [1–3]. For example, cognitive-behavioural therapy (CBT) strategies like identifying unhelpful thoughts and reframing problems, can contribute to the development of resilience or the ability to cope adaptively with stress [3,23].

The aim of this review is to examine evidence for the effectiveness of preventative early interventions to protect the well-being of military personnel coping with the pressures of adjusting from military to civilian life. Reviews of strategies including *Third Location Decompression* (TLD), *Battlemind* and *Trauma Risk Management* (TRiM) which are also preventative measures and aim to protect mental health and well-being are reviewed elsewhere, and are not covered in this review [1–3,24–26]. Some efficacy of *Battlemind* has been demonstrated in the US, though replication in the UK had mixed results, and most effects were seen on the reduction of binge drinking [26–28]. To date there is a lack of review level evidence of non-standardised preventative interventions addressing specific constructs like irritability and distress, which could form components of larger programmes, and the authors are not aware of any existing systematic reviews. To address this gap in evidence, this review seeks to evaluate the effectiveness of short preventative interventions not previously evaluated in reviews of more extensive and standardized programmes. The review considers interventions for protecting the well-being of military personnel adjusting to civilian life and therefore includes Reservists, veterans and soldiers during the post-deployment period.

It is important to explore the effectiveness of preventative interventions given the potential costs to individuals and their families if distress requires clinical intervention [6, 11, 19]. Similarly it is important to evaluate the evidence base underpinning the effectiveness of interventions in order to best inform service developments [1]. The information in the review will therefore be of interest to a number of stakeholders in military, public health and mental health settings.

## Method

The review, informed by guidelines from PRISMA (Preferred Reporting Items for Systematic Reviews and Meta-Analyses) and the Centre for Reviews and Dissemination, was registered with the International Prospective Register for Systematic Reviews (CRD42015026341) [29,30]. See S1 Appendix for the PRISMA checklist.

### Inclusion criteria

Articles were included in the review if they evaluated the effectiveness of preventative interventions to protect the well-being of military personnel adjusting to civilian life. Participants included Reservists, veterans and soldiers in the post-deployment period. Articles were included if they evaluated preventative interventions for well-being or pre-clinical distress. Inclusion criteria are summarised in Table 1. Articles evaluating interventions for specific clinical conditions, and those purposively selecting individuals with clinical diagnoses, for example, post-traumatic stress disorder (PTSD) were excluded. Studies were not included if they only focussed on spouses, parenting or children of military personnel; nor were evaluations of residential interventions including couples’ reunification retreats (for example see Davis et al. [31]).

**Table 1:** Study inclusion criteria.

**Population**	Serving or former military personnel adjusting to civilian life
**Intervention**	Brief preventative interventions promoting well-being
**Comparator**	Usual, other intervention, or none
**Outcome**	Improved psychological/emotional well-being
**Study design**	Observational/interventional

### Search strategy and information sources

Searches were conducted in the following electronic databases with a final search conducted in November 2017: MEDLINE, PsycINFO, EMBASE, Cochrane Central Register of Controlled Trials (CENTRAL), Health Management Information Consortium (HMIC), Web of Science, CINAHL, PubMed, PILOTS, PAIS International, Project Cork, Ministry of Defence (gov.uk) and the US Defence Technical Information Centre (dtic.mil). A general internet search was conducted via google.co.uk; in addition forward and backward citation searches of included articles, and hand searches of the Journal of the Royal Army Medical Corps and Military Medicine.

The search strategy included three key areas: (1) military personnel of all branches and statuses; (2) adjusting to civilian life; and (3) psychological and emotional well-being. The search syntax and search terms were adapted for use in different databases. Inclusion was limited to peer-reviewed English-language articles, and no publication date restrictions were imposed. The MEDLINE search strategy is shown in S2 Appendix.

### Study selection

Screening of titles and abstracts was performed by three researchers (AB, JF & SW). Subsequently, two researchers (AB & SW) independently assessed full-text articles for eligibility. Where disagreement about the inclusion of particular studies occurred, this was discussed with the wider team until consensus was reached.

### Data collection process and data items

A data extraction table was developed to record study characteristics, including country, population, recruitment source, intervention, comparator, study design, outcome measures, and findings. Data was extracted independently by two researchers (AB & JF).

### Risk of bias in individual studies

The Quality Assessment Tool for Quantitative Studies has evidence of reliability and validity, and was used to assess bias within studies [32,33]. Bias was rated independently by AB & JF. Where ratings fell in between categories, a lower rating was given. Disagreements about bias ratings were resolved by discussion with the wider team, and inter-rater agreement was good (kappa = .76) [34]. A summary of bias ratings is described in the results section.

### Synthesis of results

The results were synthesized narratively, as heterogeneity in the design and outcomes of included studies meant meta-analysis was not appropriate.

## Results

**Fig 1:**
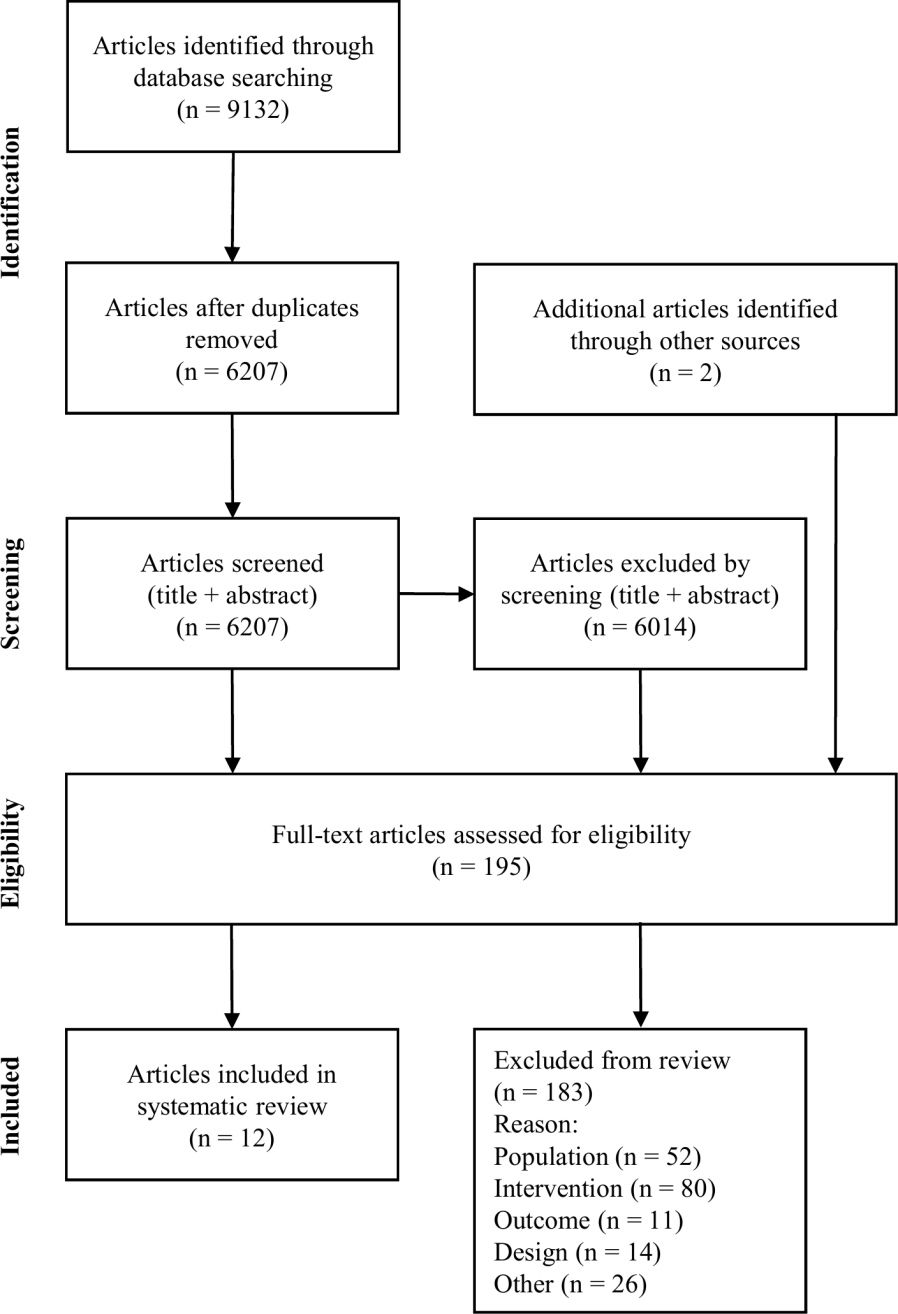
Summary of the search selection process.

Following de-duplication 6207 studies were assessed for eligibility. Twelve studies met criteria for inclusion in the review (Fig 1). All studies were from the USA.

Studies recruited soldiers recently returning from deployment [35,36], veterans of Iraq or Afghanistan [37–42], Reservists [43,44,45] and a combination of veterans and currently serving soldiers [39,46]. The majority of participants were male, with a mean age of between 18 and 37 years [35–38,40,42–45]. Two studies recruited a cohort whose mean age was between 42 and 45 years [41,46]. Interventions were for individual soldiers and veterans [37,42], and for a soldier or veteran and a family member or friend [35,39,44,46]. In order to assess the effectiveness of interventions both individual and relationship level outcomes were reported. Studies examined the effectiveness of facilitated group interventions [38,41,43,45], self-directed interventions using multi-media and online formats [39,40,44], and one study evaluated an intervention administered one-to-one by a clinician [37]. The characteristics of studies are shown in Table 2.

**Table 2:** Study characteristics.

Study (country)	Participants	Recruitment source	Intervention	Design
Interventions for Anger				
Shea, Lambert, & Reddy (2013)[37] (USA)	*N* = 23; all male; *M-age* 36 years; 91% Caucasian; 65% married; symptoms of: PTSD (30%), MDD (35%) at baseline screen; 96% employed.	Military personnel and veterans returning from Iraq or Afghanistan attending VA mental health service & having experienced ≥1 criterion A trauma while deployed, plus symptoms of anger and hyperarousal.	• Clinician led CBT intervention (specifically for reducing/modifying anger) with relaxation, arousal reduction and psycho-education. Delivered individually; 75 minutes per week over 12 weeks (*n* = 12). • Comparator: generic supportive therapy with relaxation and psycho-education (*n* = 11).	• RCT • 3-month FU
Hayes et al. (2015)[46] (USA)	*N* = 70 pairs (53% male; 90% of veterans male); *M-age* 45 years; 53% Caucasian; 72% married couples/romantic partners. 57% service connected disability; 39% deployed once; 11% currently serving.	Veterans reporting relational difficulties accompanied by romantic partner, family member, or friend; recruited via promotional material, and referrals from community veterans’ organisations, and local VA hospital.	• *Strength at Home Friends and Families*: clinician-led intervention for negative effects of trauma on relationships; involving group activities, and educational material on new behaviours and problem solving. 2 hour session per week, over 10 weeks. • Comparator: before/after.	• Cohort • 3-month FU
**Reintegration and Relationships**				
Sayer et al. (2015)[42] (USA)	*N* = 1292; 61% male; *M-age* 37 years; 64% Caucasian; 61% married; 22% mental health condition; 75% employed; average 6 years since last deployment.	Veterans self-reporting readjustment difficulties; invited to participate via register of all USA Iraq and Afghanistan veterans.	• Online expressive writing: thoughts/feelings about transition to civilian life (*n* = 508). 20 minutes/per day for 4 out of 10 days. • Comparators: factual writing (*n* = 507) or no writing (*n* = 277).	• RCT • 3 & 6-month FU
Baddeley & Pennebaker (2011)[35] (USA)	*N* = 102 couples; in 94% husband was soldier; *M-age* 32 years; 66% Caucasian; mean years married 7.5. <18 months since last deployment.	Soldiers and spouses reuniting after deployment to Iraq or Afghanistan; recruited via internet, newspaper, and radio adverts.	• Expressive writing on paper: thoughts/feelings about transition to civilian life. 3 x 15 minute sessions. • Comparator: factual writing. • 4 conditions: soldier & spouse (expressive); soldier (expressive) spouse (control); soldier (control) spouse (expressive); soldier & spouse (control).	• RCT • 1 & 6-month FU
Blevins, Roca, & Spencer (2011)[43] (USA)	*N* = 144; 92% male; *M-age* 31 years; 89% Caucasian; 62% married; ≤25% screened positive for mental health symptoms; at least one deployment; 100% most recent deployment within 3–9 months.	National Guard veterans of Iraq or Afghanistan mandatory workshop promoting readjustment. Participation in assessment was voluntary.	• *Life Guard*: clinician-led interactive workshop promoting resilience and reintegration via development of self-awareness, and goal setting (*n* = 63); 2 hour session. • Comparator: delayed intervention (*n* = 81).	• Cohort • 2-month FU
Collinge, Kahn, & Soltysik (2012)[44] (USA)	*N* = 43 couples. Veterans: 88% male; *M-age* 34 years; 86% Caucasian; symptoms of PTSD and mild depression at baseline screen (veterans & partners); 1–10 years post-deployment.	Army National Guard veterans and a significant relationship partner, recruited through post-deployment presentations and newsletters.	• *Mission Reconnect*: multi-media self-directed intervention for at home, to promote well-being and connection in relationships via stress management, relaxation, and massage. Used ≥ three times a week over 8 weeks. • Comparator: before/after.	• Cohort • 1 & 2-month FU
Kahn et al. (2016)[39] (USA)	*N* = 160 couples. Veterans: 81% male; 32% still serving (average 2 deployments); 29% PTSD symptoms; 56% Army.	OEF/OIF/OND combat deployed veterans and a significant relationship partner, recruited through social media and veteran websites.	• *Mission Reconnect (MR)*: multi-media self-directed intervention for at home over 8 weeks using techniques of mindfulness, massage, relaxation & for connection in relationships (for a minimum 40 minutes per week). • *Comparator*: weekend program or *MR +* weekend program or waitlist control.	• RCT • 2 & 4-month FU
**Resilience**				
Tenhula et al. (2014)[41] (USA)	*N* = 479; 83% male; *M-age* 42 years; 58% Caucasian.	Veterans experiencing distress or post-deployment readjustment challenges; 75 VA sites enrolled.	• *Moving Forward*: a resilience/prevention programme for readjustment challenges comprising: problem solving, and regulation of negative emotions; clinician-led with a manual; four group sessions. • Comparator: before/after.	• Cohort entry/exit
Griffith & West (2013)[45] (USA)	*N* = 441; 75% male; 49% >38 years.	Army National Guard; participants completing an army resilience training programme.	• *Master Resilience Training*: techniques to promote strong relationships, optimism, mental agility, self-awareness, self-regulation, and character strength. 4 modules taught in 1 week. • Comparator: none.	• Cross-sectional
Van Voorhees, Gollan, & Fogel (2012)[40] (USA)	*N* = 50; 90% male; *M-age* 30 years; 73% Caucasian; 45% married; 12% history of depression.	Veterans of Iraq or Afghanistan experiencing symptoms of distress and/or depression; recruited via social media, study website and online adverts.	• *Vets Prevail*: CBT-based psycho-education; online format with multi-media materials plus motivational interviewing and peer support; 6 x 0.5 hour sessions. • Comparator: before/after.	• Cohort • 1, 2, and 3-month FU
Sylvia et al. (2015)[38] (USA)	*N* = 15; 47% male; *M-age* 37 years; 80% married; 50% active duty.	Post-9/11 veterans recruited via adverts, social media and soldier/veteran mail networks.	• *Resilient Warrior*: stress management and resilience program; 4 x 2-hour weekly sessions.	• Cohort pilot/ feasibility study
**Cognitive training**				
Shipherd, Salters-Pedneault & Fordiani (2016)[36] (USA)	*N* = 1524; 90% male; *M-age* 28 years; 62% Caucasian; 8% officer rank.	Soldiers 3–12 months post-deployment recruited via post-deployment health assessments, adverts and social media.	• Brief training (60 minutes) for skills to manage intrusive cognitions by accepting them (*RESET)*. Used prompt cards and audio recordings. • Comparators: training to change intrusive thoughts; psychoeducation; psychoeducation about intrusive thoughts.	• RCT • 1-month FU

*Key*. CBT: cognitive behavioural therapy; FU: follow-up; *M-age*: mean age; MDD: major depressive disorder; OEF: Operation Enduring Freedom; OIF: Operation Iraqi Freedom; OND: Operation New Dawn; PTSD: post-traumatic stress disorder; RCT: randomized controlled trial; VA: Veterans Affairs.

### Study findings

The study findings are grouped into themes according to the aim of each intervention.

### Interventions for anger

**Fig 2:**
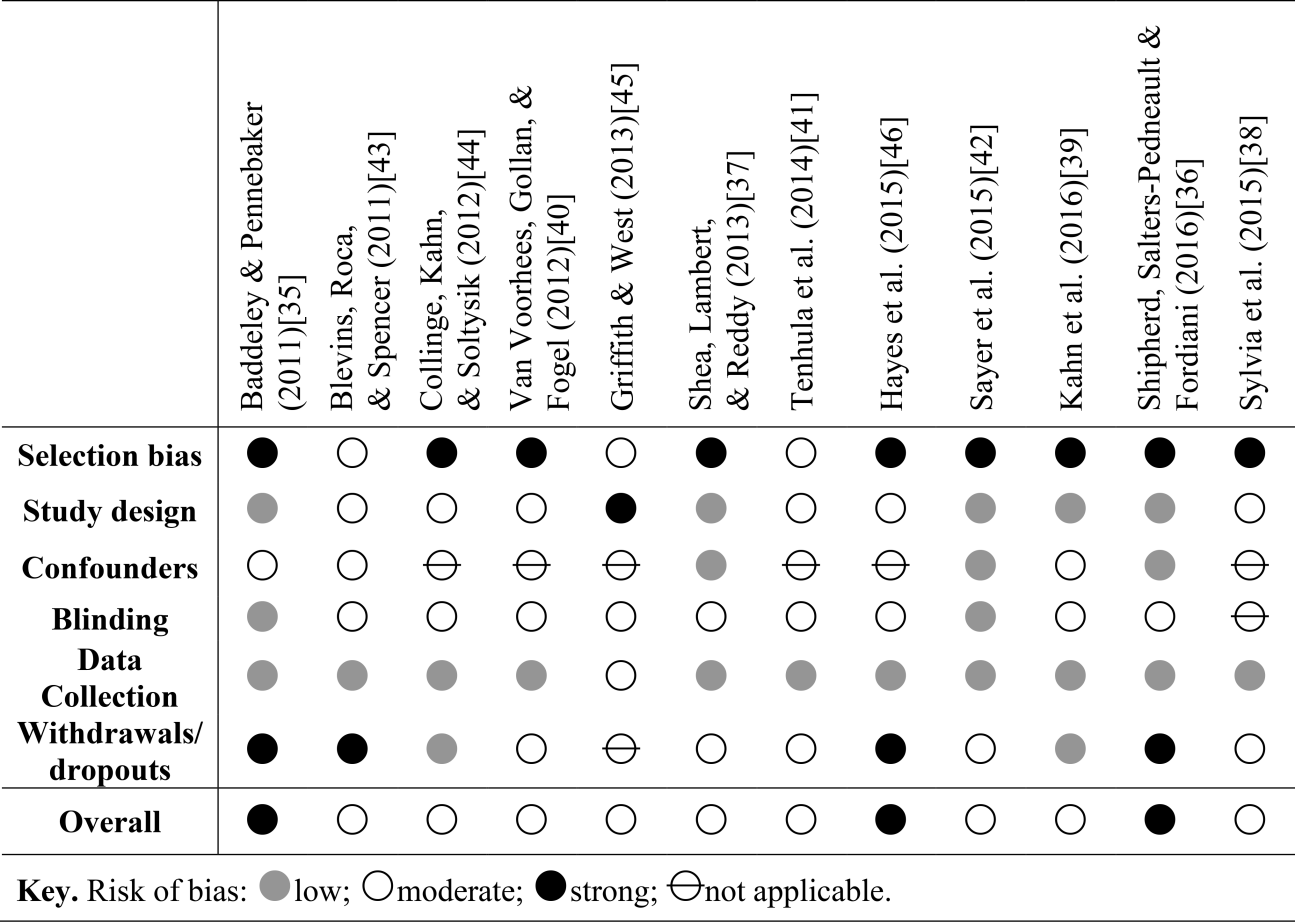
Risk of bias within studies.

The first study by Shea, Lambert & Reddy [37] evaluated an early intervention for anger with USA veterans of Iraq and Afghanistan who reported difficulties resulting from anger and hyperarousal [47,48]. Participants 96% of whom were currently employed, were recruited from a Veterans Affairs mental health service. The intervention which included cognitive restructuring, managing arousal levels, relaxation, behavioural coping strategies, imagery exposure and psycho-education was adapted for veterans and incorporated some components of *Battlemind* [27]. Compared to a control group, participants reported significant improvements with managing anger, interpersonal relationships, and social functioning. In addition, reductions in aggressive behaviour approached significance, and most of these improvements were maintained three months later. The study was a randomized controlled trial (RCT) so had a strong design and researchers measuring the outcomes of the intervention were blind to the allocation of participants to treatment or control group. However being a clinician-led, one-to-one approximately hour-long session over twelve weeks, the intervention was resource intensive. The aim of the study was to evaluate an early intervention before any secondary problems of anger occurred; however participants were recruited from a clinical service, and up to 35% of participants were found to have symptoms of PTSD or major depressive disorder (MDD). Given this, generalisability of the findings to individuals without mental health symptoms cannot be assumed. A summary of bias ratings within studies is shown in Fig 2.

In the second study Hayes et al. [46] evaluated *Strength at Home Friends and Families*, a preventative group intervention addressing negative consequences of trauma-related anger and aggression on relationships. As in the previous study [37] the aim of the intervention was to relieve relationship conflict before there were severe consequences, and with couples demonstrating physical aggression screened out the main difficulties were psychological aggression. Led by a clinician and delivered to groups of between three and five veterans (and a partner), it involved learning new behaviours, problem-solving and exploring ways to change. There were ten weekly sessions each two hours long, and a follow-up assessment three months after the intervention. Seventy veterans and a significant other (a romantic partner, family member or friend) reporting relationship difficulties were recruited via adverts and veteran’s organisations in the USA. 34% of participants had served in Iraq or Afghanistan, and mean age was 45 years. The majority of participants (53%) had served in the Army, 11% were currently serving, 39% had been deployed once and 21% three or more times. Participants were recruited from a community setting, and although individuals with mental health conditions and substance use disorders were excluded, symptoms of PTSD and depression were measured by the authors during baseline screening, plus 36% of participants reported being discharged from the military with a severance or disability payment. Participation in the intervention was associated with significant reductions in psychological aggression, and symptoms of depression and PTSD. Improvements occurred for veterans and their partners, and apart from depression symptoms, effects were maintained three months later. Partners of veterans (but not veterans) also reported significant improvements in relationship adjustment. The intervention did not have a significant effect on physical aggression (rates being low at baseline) or perceived social support [46]. Attrition from the study was high (only 63% completing the intervention and 57% remaining for the follow-up assessment), in addition limitations in the study design included the lack of a control group, or any assessment of treatment fidelity. The study findings are shown in Table 3.

**Table 3:** Study findings.

Study	Outcome measures	Findings
**Interventions for Anger**		
Shea, Lambert, & Reddy (2013)[37]	• Overt Aggression Scale-Modified[49] • State-Trait Anxiety Inventory-2 (Anger Expression Index)[50] • Dimensions of Anger Reactions[51] • Outcomes Questionnaire[52]	• Intervention participants: significant improvements in interpersonal/social functioning, and reductions in anger post-intervention compared to control group; effects on aggressive behaviours approached significance (large effect sizes: 0.78–1.22). No between-group differences in distress. Changes mostly maintained at 3-months FU.
Hayes et al. (2015)[46]	• Revised Conflict Tactics Scale (Psychological Aggression & Physical Assault subscales)[53] • DAS[54] • Quality of Relationship Inventory[55] • PHQ[56] • PTSD Checklist-C/M[57]	• Significant reductions pre- to post-intervention in psychological aggression, depression and PTSD symptoms in veterans and significant others. Effects on psychological aggression and PTSD maintained at 3-months FU. • Significant improvements in relationship adjustment reported by partners (not significant for veterans). • Significant correlations between veterans and partners in levels of psychological & physical aggression across time points. No significant change in physical aggression (from low levels at baseline) or perceived support.
**Reintegration and Relationships**		
Sayer et al. (2015)[42]	• PTSD Checklist-M[58] • BSI-18[59] • BSI (Hostility subscale)[60] • PILL[61] • Military to Civilian Questionnaire[62] • Deployment Risk and Resilience Inventory (Social Support Scale)[63] • Satisfaction With Life Scale[64]	• Compared to factual writing, expressive writing associated with significantly more reductions in anger, physical complaints, and by 6 months distress; no significant difference in PTSD symptoms, reintegration difficulty, social support or life satisfaction. • Compared to no writing, expressive writing associated with significantly more positive effects on all indicators (apart from life satisfaction). • Compared to no writing, expressive writing associated with significantly reduced odds of clinical distress; and compared to both control groups significantly reduced odds of PTSD and increased odds of being employed.
Baddeley & Pennebaker (2011)[35]	• Relationship Assessment Scale[65] • PHQ[66] • PILL[61]	• Expressive writing by soldiers (but not spouses) associated with couples reporting greater marital satisfaction at 1-month FU. Expressive writing was more beneficial to marital satisfaction where the soldier had more combat exposure.
Blevins, Roca & Spencer (2011)[43]	• SF-12[67] • PHQ-9 (Depression subscale)[56] • GAD-7[68] • Brief PHQ (Panic screen)[66] • PTSD Checklist-C[69] • Buss-Perry Aggression Questionnaire[70] • DAS (short form)[71] • Conflict Tactics Scale[72] • AUDIT[73]	• After 2 months the intervention group demonstrated significant reductions in symptoms of depression, PTSD, GAD and relationship satisfaction. No significant changes for the control group on any measure. Significant between-group differences in depression symptoms and relationship satisfaction.
Collinge, Kahn & Soltysik (2012)[44]	• PTSD Checklist-C[69] • BDI II[74] • Perceived Stress Scale[75] • Compassionate Love Scale[76] • Self-Compassion Scale[77] • Quality of Life Inventory[78]	• For veterans and partners significant reductions in symptoms of PTSD and depression at 2-months FU; significant improvement in self-compassion 1-month FU (approaching significance 2-months FU). For partners (but not veterans) significant reductions in perceived stress at 2-months FU. After massages soldiers reported significant reductions in physical pain, tension, irritability, anxiety, worry, and depression; in addition a significant decline over time in pre-massage tension and irritability.
Kahn et al. (2016)[39] (USA)	• Perceived Stress Scale[75] • BDI II[74] • PTSD Checklist-C[69] • Self-Compassion Scale[77] • Response to Stressful Experiences Scale[79] • Multidimensional Scale of Perceived Social Support[80] • Pittsburgh Sleep Quality Index[81] • Dyadic Adjustment Scale[82]	Significant improvements (at 4-months follow-up) in PTSD symptoms, stress, self-compassion and depression for veterans in the *Mission Reconnect* group; and in stress and self-compassion for partners. • At 4 months most significant difference between MR participants and waitlist control (stress, self-compassion, pain). • Significant effects for veterans and partners after massage for pain, tension, irritability, anxiety and depression.
**Resilience**		
Tenhula et al. (2014)[41]	• PHQ-9[56] • Outcomes Questionnaire-30[52] • Brief Resilience Scale[83] • Social Problem Solving Inventory-Revised: short form[84]	• Significant improvements pre/post-intervention on measures of depression, social problem-solving and resilience.
Griffith & West (2013)[45]	• Online questionnaires devised by the authors measuring resilience competency skills, stress, worry and anxiety	• Participants reported improvement across measures of resilience. • All outcomes negatively correlated with worry and anxiety; regression analyses did not indicate strong stress buffering effects of the training.
Van Voorhees Gollan, & Fogel (2012)[40]	• Centre for Epidemiological Studies-Depression Scale-10[85] • PTSD Checklist-M[86] • SF-12[87]	• Significant decline in depression and PTSD symptoms by 3 months.
Sylvia et al. (2015)[38]	• PHQ[56] • GAD-7[68] • Perceived Stress Scale[75] • General Self efficacy Scale[88] • Resilience Scale[89]	• Pre to post-intervention changes significant for symptoms of depression, perceived stress; marginally significant for anxiety and self-efficacy. No significant change for resilience. • Good acceptability reported.
**Cognitive training**		
Shipherd, Salters-Pedneault & Fordiani (2016)[36]	• Experience of Intrusions Scale[90] • PTSD Checklist-C[69] • Depression Anxiety Stress Scales[91] • Expectancy of Therapeutic Outcome Questionnaire[92] • WRAIR[27,93]	• *RESET* group: significantly more reductions relative to controls in intrusive cognitions, PTSD, depression and anxiety (small/medium effect).

Key. AUDIT: Alcohol Use Disorders Identification Test; BDI: Beck Depression Inventory; BSI: Brief Symptom Inventory; DAS: Dyadic Adjustment Scale; FU: Follow-Up; GAD: Generalised Anxiety Disorder scale; PHQ: Patient Health Questionnaire; PILL: Pennebaker Inventory of Limbic Languidness; PTSD Checklist-C/M (civilian/military); SF 12: Short Form Health Survey; WRAIR: Walter Reed Army Institute of Research Deployment Experiences Scale.

### Interventions for reintegration to civilian life and relationships

Two studies evaluated the effectiveness of expressive writing about the feelings associated with the challenges of reintegrating back into civilian life and relationships. In the first study by Sayer et al. [42] participants (*N* = 1292) who reported experiencing reintegration difficulties were recruited via a register of all USA Iraq and Afghanistan veterans. Large numbers invited to participate were excluded as they did not report reintegration difficulties. Half of those recruited had a baseline positive PTSD screen and 29% a mental health clinic visit in the past three months. The study had a strong design (RCT) and data analysts were blind to the intertvention or control group allocation of participants. Compared to writing about facts (with no emotional content), or not writing at all, soldiers in the expressive writing group (which involved writing online for twenty minutes a day for four days during a ten day period) reported some significant reductions in anger, physical symptoms and by six months distress. There were improvements across more indicators and effects sizes were greatest when expressive writing was compared to no writing, rather than to factual writing. On average only two or three sessions were completed (out of four) [42].

In a second RCT by Baddeley and Pennebaker [35], the effectiveness of expressive writing (on paper during three fifteen minute sessions) about emotions surrounding the transition home by soldiers and their spouse was evaluated. Couples (*N* = 102) reuniting post-deployment, where the soldier had returned from Iraq or Afghanistan in the previous eighteen months, were recruited via media and internet adverts. Couples reported greater marital satisfaction one month later if the soldier engaged in expressive compared to non-emotional writing, though this was not the case when the spouse engaged in expressive writing, and effects were greatest for those couples with a soldier reporting higher combat exposure.

*Life Guard* is an intervention developed by the researchers Blevins et al. [43] and informed by CBT and Acceptance and Commitment Therapy (ACT) [94]. The two-hour long single-session intervention aims to promote resilience and reintegration through the acquisition of skills, including self-awareness, psychological flexibility, normalisation of maladaptive thoughts, acceptance, mindfulness and commitment to values and goals. Blevins et al. [43] evaluated the intervention in a study conducted in Arkansas, (where participation in *Life Guard* was mandatory for National Guard), with soldiers (*N* = 144) with at least one deployment to Iraq or Afghanistan. Study attrition was high and the findings require replication by an independent research group; however, post-intervention assessments indicated significant reductions in symptoms of depression, generalized anxiety disorder and PTSD over time. In addition positive changes in depression symptoms and marital satisfaction were more significant in those receiving the intervention than in participants in the control group.

*Mission Reconnect* is a stress management and relaxation intervention to promote connection in relationships. Couples were recruited to a feasibility study by Collinge, Kahn, & Soltysik [44] via adverts and Army National Guard post-deployment events. Veterans and their partners reported significant improvements in self-compassion one month after the intervention, and PTSD and depression symptoms two months later. Partners (but not veterans) reported significant reductions in perceived stress two months later. Veterans reported significant improvements in physical pain, tension, irritability, anxiety, worry and depression after the intervention, and a significant decline over time in pre-session tension and irritability [44]. *Mission Reconnect* is self-directed and multi-media, thus could be administered at home. Encouragingly, some intervention effects were maintained two months later, and acceptability was high; however, the lack of a control group compromised the study design [44]. Additionally although the intervention was a wellness programme (rather than a mental health treament) PTSD symptoms in participants were shown at baseline, so generalisability to less symptomatic groups cannot be assumed.

In a follow up to the feasibility study by Collinge et al [44], Kahn et al. [39] evaluted *Mission Reconnect* with post-9/11 veterans and partners recruited via the internet and social media adverts. The intervention was found to be acceptable to recipients and attrition was low, though participants did self-select into the study. Significant improvements in well-being were seen in the group receiving the intervention at the two and four months follow-up as measured on a number of indicators including stress, depression, PTSD symptoms and self-compassion. The differences in improvements were most significant when MR was compared to the waitlist control group rather than to the other (weekend) intervention group [39].

### Resilience interventions

Four studies evaluated interventions for promoting resilience. In the first study Tenhula et al. [41] examined a preventative programme for veterans experiencing readjustment challenges called *Moving Forward* [95]. This focussed on problem-solving skills and regulation of negative emotions and techniques included positive visualisation, externalisation, and managing arousal levels. Participants experiencing distress or challenges reintegrating into civilian life were recruited via Veterans Affairs with most having served in Iraq or Afghanistan and had a mean age of 42 years. The aim of the intervention was the prevention of mental health conditions, though self-report baseline screening measures indicated the presence of some symptoms including of depression. At the end of the intervention, participants reported significant reductions in symptoms of depression and distress, and improvements in social problem-solving and resilience. In addition, acceptability of the intervention to participants was good; however, the lack of a comparison control group compromised the study design [41].

In the second study by Griffith & West [45], Army National Guard soldiers who had completed the USA Army *Master Resilience Training* [96] were assessed for the acquistion of skills. Almost half of the participants were over 38 years old and more than 70% were sergeants or held a higher rank. The training focussed on developing six competencies: connection, optimism, mental agility, self-awareness, self-regulation, and character strength. Participants reported improvements in all competencies, and the responses from participants indicated acceptability of the intervention with more than 90% stating that they used the skills in both military and civilian life. Resilience competencies were negatively correlated with worry and anxiety; however, a regression analysis did not indicate a strong stress-buffering effect of the training. A limitation of the study was the lack of standardized outcome measures and the cross-sectional design, so there was no assessment of whether improvements were sustained over time [45].

The third intervention, *Vets Prevail*, evaluated by Van Voorhees, Gollan & Fogel [40] had a trans-diagnostic approach to address early signs of distress. Individuals with any significant psychiatric history were excluded, as were those with too few self-reported symptoms (including scoring below the population mean for depression). Participants were veterans who had served in Iraq or Afghanistan in the last five years, and were recruited via online adverts and social media. The intervention consisted of six half-hour sessions teaching coping techniques, such as behavioural activation and problem-solving, in addition to online motivational interviewing, and peer-to-peer counselling via instant messaging. Participants attending *Vets Prevail* reported significant reductions in symptoms of depression and PTSD three months later. However, given participants self-selected into the study, generalisability of the findings to other individuals who may be less motivated cannot be assumed; and in addition the lack of a control group was a design limitation [40].

Finally Sylvia et al. [38] conducted a pilot feasibility study of a stress reduction and resilience training program with participants recruited through adverts and social media. Although a measure of resilience did not indicate significant change after the intervention, measures of depression and perceived stress demonstrated significant improvements. Acceptability of the intervention was good though being a pilot study numbers recruited were small (*N* = 15) and there was no comparator group.

### Cognitive training

Shipherd, Salters-Pedneault & Fordiani [36] evaluated a brief intervention called *RESET* for managing intrusive cognitions by accepting emotions and thoughts, with soldiers (*N* = 1524) recently returned from deployment. The intervention had some small beneficial effects on reducing distress and impairment from intrusive thoughts, compared to three control groups including an intervention aimed at changing thoughts. The study had a strong design (RCT) however self-selection of participants into the study and the high attrition rate (only 46% completing at follow-up) may have biased the findings.

## Discussion

### Key findings

To our knowledge, this is the first systematic review to examine the effectiveness of preventative interventions for well-being which are either not standardized or routinely implemented across services for veterans and military personnel adjusting to civilian life. Few studies met inclusion criteria, and all were conducted in the USA. The review has a different focus to previous reviews of standardized programmes for stress and risk management such as *Battlemind* and *TRiM* in the UK, which could be described as well-being interventions, but are implemented at platoon or unit level [24–27].

The studies in the review evaluated the effectiveness of preventative interventions for managing anger, connection in relationships, intrusive cognitions and resilience. Interventions were for individuals, couples and groups, and delivered face-to-face or online. The findings of the individual studies demonstrated some evidence of effectiveness of preventative interventions like expressive writing on a number of indicators of well-being for military personnel adjusting to civilian life.

There was considerable heterogeneity across the studies reviewed in terms of design, outcome measures used and the interventions evaluated, so that generalisability of the findings beyond individual studies cannot be assumed. Studies in the review included the recruitment of participants of different rank, branch of service, and conflict era, including those serving in the Persian Gulf and Vietnam Wars. There was therefore considerable between-study variation in the characteristics of participants, in addition information on the number of previous deployments, percentage of those currently serving, and their rank or branch of service was not always described. The pressures on individuals and impact on well-being will vary as a function of these differences, for example for those still serving compared to those who have left, with research on transition and adjustment to civilian life indicating that leaving the service may be a challenge to well-being [4,5].

The majority of participants recruited to the studies reviewed were male (more than 75%) apart from the study by Sayer et al. [42] in which 39% were women and in the pilot study (*N* = 15) by Sylvia et al. [38] recruiting 53% women. Sayer et al. [42] was also the only study that specifically examined differential effects of a well-being intervention (expressive writing) according to gender, finding lower odds of distress in women but no gender/condition interaction. Therefore there were few women represented in the evaluations of the well-being interventions. Other between study variations included when the intervention was delivered for example in the study by Griffith and West [45] study participants were National Guard soldiers and received the (resilience training) intervention during their work. In contrast in the study by Baddeley and Pennebaker [35] participants had self-reported difficulties and chosen to receive the intervention (expressive writing). These are quite different recruitment methods and scenarios that have implications for intervention acceptability, motivation to engage and attrition and may have biased the findings of the individual studies [42]. Another between-study variation in the characteristics of participants was whether they were currently serving soldiers, Reserves or veterans. For example two studies evaluated expressive writing including Sayer et al. (2015) [42] who recruited veterans, and Baddeley and Pennebaker [35] who recruited recently deployed soldiers. Both studies were RCTs and so had strong designs, however the study recruiting veterans (who were last deployed on average six years prior to the study) [42], measured more effectiveness across more indicators of well-being than the study recruiting currently serving soldiers [35]. These are very different groups and the findings suggest the expressive writing intervention was more effective with veterans though replication would be required. This highlights the diversity of the participants recruited to the studies and how their needs will differ depending on their circumstances, for example Reserves have been found to face different challenges compared to those serving as Regular soldiers [97]. The effectiveness of the well-being interventions demonstrated in the individual studies would therefore need to be replicated in diverse groups of military personnel, which captured differences relating to country, gender, and service setting.

An inclusion criteria for the review was that studies evaluated preventative well-being interventions, and studies that purposively recruited individuals with specific clinical conditions, for example PTSD or traumatic brain injury, were excluded. In addition the studies in the review evaluated interventions aimed at alleviating sub-clinical difficulties; nevertheless, screening measures taken in the studies at baseline indicated the presence of mental health symptoms in many participants. For example in the study by Blevins et al. [43], participants were currently serving National Guard, and 25% had symptoms of generalized anxiety disorder. Also in many cases participants were recruited because (sub-clinical) difficulties including relationship problems, hyperarousal, or difficulties adjusting to civilian life had brought them to attention of services. In the study by Shea et al. [37] which evaluated an intervention designed to address difficulties (related to anger) before any secondary effects occurred, participants were recruited from mental health services and up to 35% had symptoms of PTSD or Major Depressive Disorder. The effectiveness of the well-being interventions therefore may not necessarily be transferrable to individuals without mental health symptoms and would need to be evaluated with symptom free groups separately. For example in the study recruiting recently deployed soldiers the most beneficial effects were seen with those with high combat exposure [35].

This highlights the issue of whether preventative interventions are most useful for individuals with no symptomatology, those with some sub-clinical symptoms or both. As well-being is a continuum, focussing on prevention could avert more severe symptoms when a person has a clinical diagnosis, and if implemented at primary care level could potentially offset the need for more expensive secondary or tertiary level care. Investing in well-being interventions upstream of clinically commissioned health services may be beneficial if they protect mental health, however this requires proof of effectiveness and this may be easier to demonstrate in individuals with some symptoms.

A number of the interventions were shown to benefit the support networks of military personnel for example by reducing symptoms of PTSD and depression in partners, and improving indicators of relationship quality such as marital satisfaction [35,44], and in some cases, positive effects were greater for partners than for soldiers [46]. The inclusion of significant others in interventions for soldiers’ well-being may be important, given the unique pressures experienced by military spouses, and the support families provide when military personnel return home [15,98].

### Strengths and limitations of the review

A small number of studies met criteria for inclusion in the review; and the heterogeneity in outcome measures, participants and interventions evaluated meant studies were not sufficiently similar to pool the results and make any statistical estimates of effects across independent studies. The different characteristics of participants and with all of the studies being from the USA, meant that generalisability to other countries and diverse military groups cannot be assumed, given the differences in culture, military structures, health, and social care services; for example the different definitions of a veteran in different countries [99, 100]. A strength of this review is that it considers the effectiveness of preventative interventions like expressive writing, which may form part of more extensive programmes, in which some components (and it may not be clear which ones) may be more effective than others [28,101]. Additionally, the current review evaluated interventions implemented with Reservists, veterans and those in the post-deployment period.

### Implications and areas for future research

The review highlights three main issues regarding well-being interventions for military personnel adjusting to civilian life that would be important to consider in future research and service development. These include a conceptualisation of well-being for military groups transitioning to civilian life, the acceptability of well-being interventions which may be perceived as treatment, and replication of the evidence of effectiveness with diverse groups of participants.

In future studies it would be important to examine what well-being means to military personnel adjusting to civilian life, and this could be done using qualitative research that explores the best definition of well-being for this group. A clear conceptualisation of well-being would facilitate the development of reliable and valid outcome measures which are key to demonstrating the effectiveness of preventative interventions. The studies in the review mainly used measures of mental health to assess the effectiveness of well-being interventions, however when there is a focus on indicators of distress, the more positive qualities of well-being may not be identified and in future studies it would be important to explore positive conceptualisations of well-being from the perspective of military personnel transitioning to civilian life [12,102,103].

Although the acceptability of the interventions to those receiving them was good (when measured), attrition was high in a number of the studies reviewed, and further exploration of the best conceptualisations of preventative interventions for military personnel adjusting to civilian life would be important in future research. For example skills/strength training may be more acceptable than interventions presented as treatment [3,37,94]. Future qualitative research with a range of stakeholders could explore the acceptability of interventions like expressive writing for a population group who value mental toughness, and also how and if well-being interventions could be reconciled with a paradigm of stoicism and hardiness is an important area for future research [104–107]. In future research it would be important to consider gender in evaluations of interventions for well-being because distress may not present in the same way in men and women [105,106].

The findings suggest that in future research it would be important to replicate the evidence of effectiveness in the individual studies reviewed using robust designs for example RCTs with adequate statistical power, and prospective data collection to evaluate how long any effects last. Future studies should also examine how and when low well-being should be identified [89], for example evidence of the effectiveness of mental health screening in military populations varies across countries and was not found to be helpful in the UK [108–110]. In the studies reviewed many participants had come to the attention of services as they were experiencing difficulties or had an interest that prompted them to elect to receive the intervention.

## Conclusions

In conclusion the preventative interventions reviewed demonstrated some potential to improve indicators of well-being in soldiers and veterans adjusting to civilian life. Most of the participants in the studies reviewed were veterans and it was in this group that most evidence of effectiveness was seen. Although the aim of the interventions evaluated in the review was to protect well-being by addressing pre-clinical difficulties, the most effectiveness was demonstrated with veterans who had some mental health symptoms at baseline, and who were recruited via clinical services or elected to receive the intervention. Given this the generalisability to symptom free groups cannot be assumed. Whether the interventions could be incorporated into exit programmes which prepare for the transition to civilian life with symptom free groups would need to be evaluated in future research and it may be that the interventions as currently stand are most effective for those expressing difficulties and/or who have left military service.

In conclusion the review findings suggest that overall the well-being interventions evaluated may have the most benefit for veterans who report experiencing difficulties adjusting to civilian life, and that further evaluation of effectiveness in large trials with diverse groups, and exploration of acceptability and concepts of well-being is required. The study findings will be of interest to a number of stakeholders in military, public health and mental health settings.

## Supporting information

S1 Appendix PRISMA checklist. (DOCX)

S2 Appendix Medline search strategy. (DOCX)
