# Supplementary material for: Is prevention better than cure? A systematic review of the effectiveness of well-being interventions for military personnel adjusting to civilian life
Source: PLoS One. 2018 May 2;13(5):e0190144. doi: 10.1371/journal.pone.0190144 (PMC5931793; doi:10.1371/journal.pone.0190144)
Supplement: S2 Appendix — (DOCX) [file pone.0190144.s002.docx]

**S2 Appendix.** **Medline search.**

| 1. exp military personnel/ or exp veterans/ 2. (army or navy or marine? or ((air or armed or special) adj1 force?) or military or paramilitary or reservist or reserves or (reserve adj1 unit?) or veteran? or soldier? or conscript? or cadet? or active-duty or (active adj1 duty)).mp. 3. (servicem?n or servicewom?n or servicemember? or ((coast or national) adj1 guard) or ex-service* or (service adj1 (personnel or leaver?)) or ((reserve or regular) adj1 force?)).mp. 4. or/1-3 5. (transition* or adjust* or readjust* or re?integrat* or civilian).mp. 6. (discharge? or post?discharge or post?deployment or post?conflict or post?combat or adapt* or re?establish* or re?entry or re?enter* or re?employ* or returning).mp. 7. ((coming adj2 home) or (return* adj9 (veteran? or deployment or military))).mp. 8. ((Cope or coping) adj5 (discharge or transition* or military)).mp. 9. (separated or separate?).mp. 10. or/5-9 11. "Quality of Life"/ 12. Stress Disorders, Post-Traumatic/ 13. Combat Disorders/ 14. exp mental disorders/ 15. veterans health/ 16. exp mental health/ 17. exp military personnel/px 18. exp veterans/px 19. (anxiety or stress or depression or ptsd or resilience).mp. 20. ((mental or psychological) adj2 (ill* or disorder? or health or (well?being or (well adj1 being)))).mp. 21. ((coping or social) adj2 behavio?r).mp. 22. (well?being or (well adj1 being)).mp. 23. ((psychological or behavio?ral) adj2 problem*).mp. 24. "quality of life".mp. 25. or/11-24 26. Marine Biology/ or Marine Toxins/ or exp Aquatic Organisms/ 27. humans/ 28. animals/ 29. 28 not (27 and 28) 30. (4 and 10 and 25) not (26 or 29) 31. limit 30 to English language |
| --- |
